# Supplementary material for: MetaRibo-Seq measures translation in microbiomes
Source: Nat Commun. 2020 Jun 29;11:3268. doi: 10.1038/s41467-020-17081-z (PMC7324362; doi:10.1038/s41467-020-17081-z)
Supplement: Supplementary file 10 — Supplementary Data 7 [file 41467_2020_17081_MOESM10_ESM.zip › File2/Confidence_High_Taxonomy/270673_out.krona.html]

Javascript must be enabled to view this page.

members
magnitude
magnitudeUnassigned
count
unassigned
taxon
rank

270673\_out

3


SRS098717\_contig\_number\_contig-100\_25520.78245SRS144362\_contig\_number\_contig-100\_2448.240959
2

1
2
superkingdom

1
phylum
1239

1
186801
class

1
186802
order

family
31979
1

1649459
genus
1

1

SRS144362\_contig\_number\_2550
154046
species
